# Venous hemangioma arising within the nailbed: A rare clinical presentation

**DOI:** 10.1016/j.jdcr.2024.02.038

**Published:** 2024-04-09

**Authors:** Bridget Myers, Boya Abudu, Ilana Breen, Lawrence S. Chan, Maxwell Fung, Jayne Joo

**Affiliations:** Department of Dermatology, University of California, Davis, Davis, California

**Keywords:** subungual pigmented lesion, venous hemangioma, venous lake

## Introduction

Venous hemangiomas or venous lakes are acquired venous ectasias. They were first described by Bean and Walsh in 1956.^1^ They consist of dilated thin-walled superficial venules surrounded by a thick layer of fibrous tissue within the superficial dermis.^2^ These benign cutaneous findings are usually observed in older patients on sun-exposed skin.^3^ They most frequently occur on the lip, particularly the lower lip. Other reported locations include the cheeks, nose, ears, hands, and mucous membranes. They often present as sharply demarcated, dark blue, round, and smooth compressible papules.^3^ These lesions often range in size from 2 to 10 millimeters.^4^ The mechanism of development is thought to occur as a result of photodamage to surrounding dermal elastic tissue. Once damaged, the faulty dermal elastic tissue is unable to prevent the dilation of nearby venules. Lesions are often asymptomatic; however, pruritus or tenderness is occasionally observed.^5^ Patients may desire treatment because of frequent bleeding or cosmetic unacceptability.^6^ Here, we present a case of a venous hemangioma arising in an atypical location — the nailbed.

## Case report

**Fig 1:**
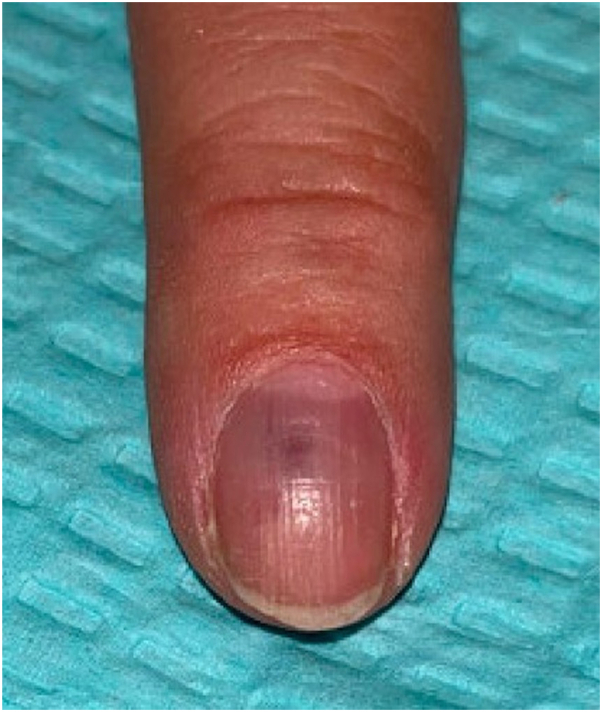
On examination, a 2 × 2 mm ill-defined subungual *dark bluish* discoloration with a violaceous hue on the central proximal portion of the nail plate of the left second finger was observed.

**Fig 2:**
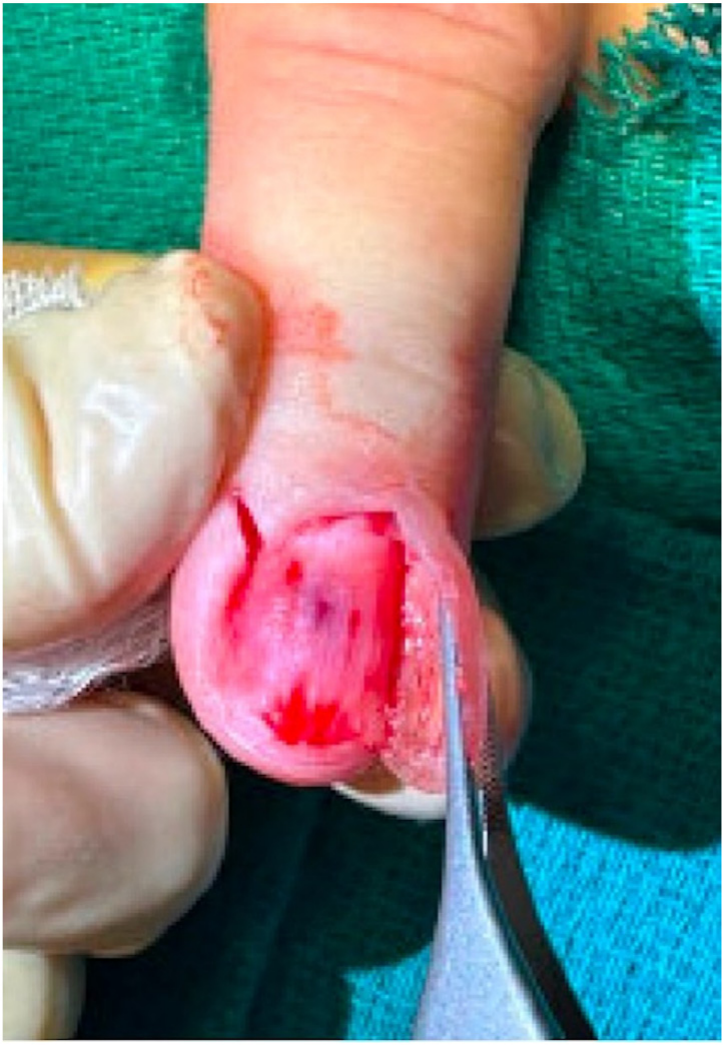
During the nail biopsy, the nail plate was elevated revealing subtle nailbed fullness surrounding a dark blue discoloration.

**Fig 3:**
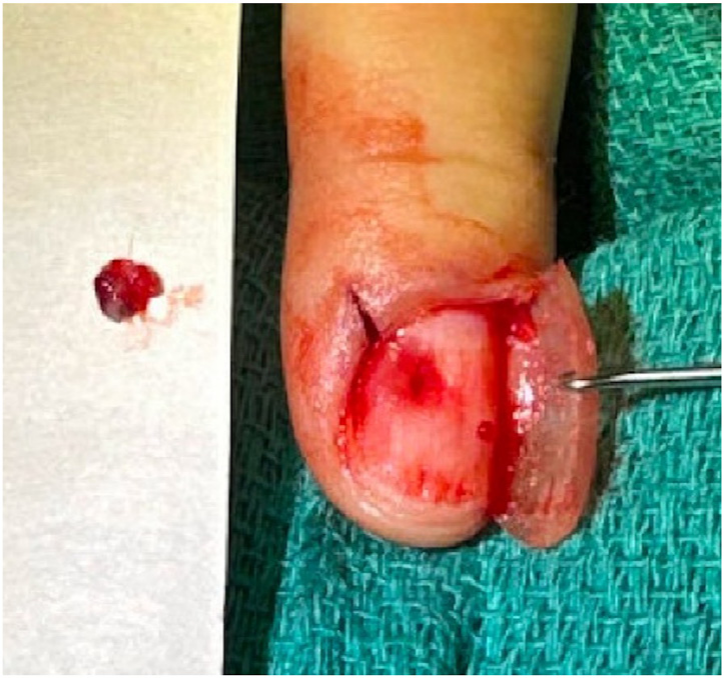
A 2 × 2 mm vascular lobule was removed and sent for pathologic examination.

**Fig 4:**
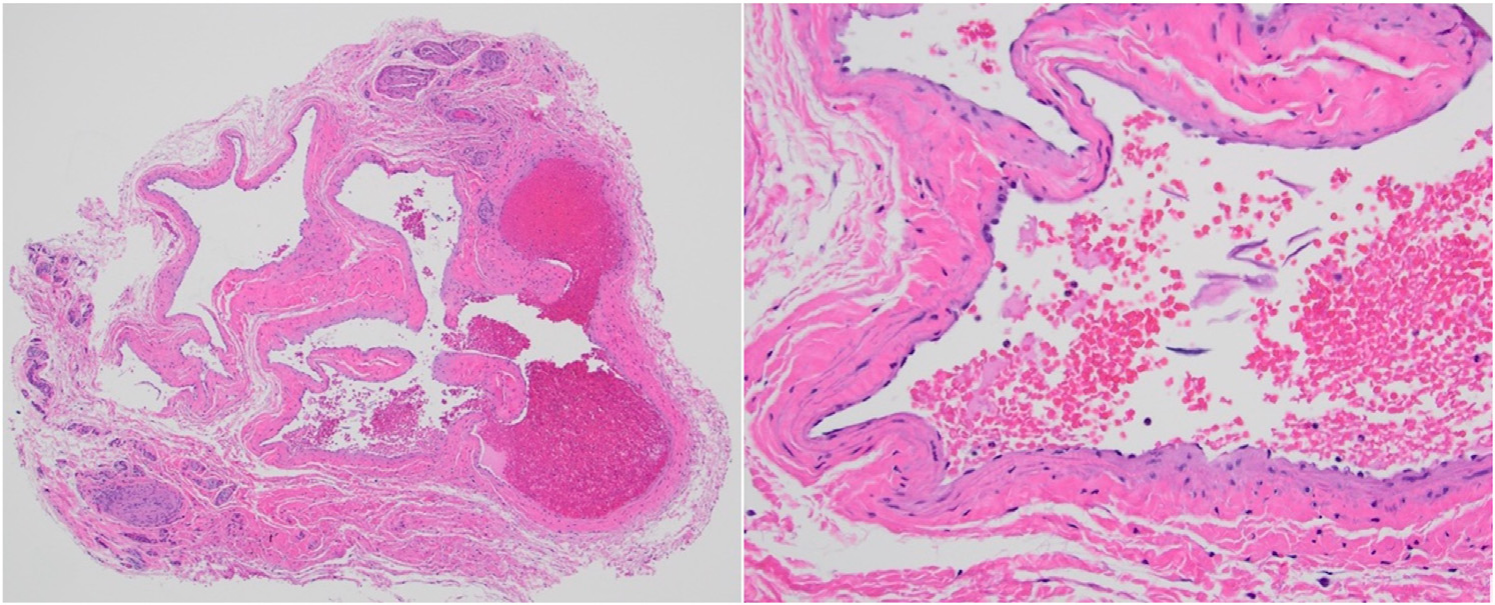
Histopathologic examination on low power (40× magnification, *left*) and high power (200× magnification, *right*) showed dilated endothelial-lined vessels within the deeper dermis, consistent with a venous hemangioma.

Our patient is a 65-year-old female who presented to dermatology due to an enlarging, dark blue-colored macule on her left second fingernail. The patient first noticed the lesion 2 years before presentation. For the past 6 months, she endorsed worsening swelling and mild pain sometimes triggered by temperature changes. She denied any bleeding, surrounding redness, or change in color. On exam, a 2 × 2 mm ill-defined subungual dark blue discoloration was noted underneath the central proximal portion of the nail plate of the left second finger (Fig 1). The nail plate was slightly deformed, demonstrating a subtle clubbed contour possibly representing mass effect due to the expanding subungual lesion. The discoloration was isolated to the nail bed and did not involve the proximal or lateral nail folds. Based on presentation, the differential diagnosis of a glomus tumor was favored versus a melanocytic neoplasm, blue nevus, pyogenic granuloma, Kaposi sarcoma, or angiolymphoid hyperplasia with eosinophils. Given the significant duration and increasing size, a nail biopsy was performed. During the procedure, the nail plate was elevated revealing subtle nailbed fullness at the site of dark blue discoloration (Fig 2). A small elliptical excision of the nailbed was performed. A 2 × 2 mm vascular lobule was dissected off the distal phalanx and excised (Fig 3). Histopathologic examination showed dilated endothelial-lined vessels within the deeper dermis, consistent with a venous hemangioma (Fig 4).

## Discussion

In this article, we present a case of a venous hemangioma arising within the nailbed. Although venous hemangiomas are common, often arising on the lip, ear, or face,^4^ this is the first reported case of a venous hemangioma arising subungually. For the dermatologist, subungual pigmented lesions frequently present a diagnostic challenge. This is due to a broad list of possible etiologies (Table I). Additionally, obstructed visibility from the nail plate and reluctance to proceed with nail biopsy due to fear of pain and risk of nail dystrophy may limit diagnostic assessment. The evaluation of subungual pigmented lesions should include careful history taking. This includes querying about trauma, athletic activities, and pertinent medications such as blood thinners.^7^ Additional tools which can assist in determining the etiology of subungual pigmented lesions include dermoscopy and ultrasound.^8^^,^^9^ On ultrasound of a subungual venous hemangioma, one could expect to see a compressible hypoechoic venous space with slow flow. Magnetic resonance imaging (MRI) can be useful if clinical or sonographic findings are inconclusive.

**Table I:** Common causes of round or oval subungual pigmentation^7^

Differential for round or ovaloid subungual pigmentation
• Subungual hematoma • Glomus tumor • Subungual lentigox • Subungual nevus • Subungual melanoma in situ • Subungual malignant melanoma • Drug-induced pigmentation o Seen with chemotherapeutic agents, antibiotics, and antimalarials • Exogenous pigmentation o Seen with hair dye, henna tattoo, tobacco, silver nitrate, or chemical agents • Onychomycosis nigricans • Venous hemangioma • Other vascular malformations

From our own experience, unlike subungual hematomas, subungual venous hemangiomas have a fairly well-circumscribed border and uniform color. On dermoscopic examination, venous hemangiomas are composed of red or blue structureless globules.^10^ In comparison, brown lines and melanin granules are characteristic of melanocytic lesions.^7^ Of note, authors Braun et al suggested that using ultrasound gel as an immersion medium for dermoscopic evaluation of nail units permits easier visualization. This is due to improved cohesion between the dermatoscope and nail plate, which limits rolling of your device. Varying the focus is also reported to improve dermoscopic evaluation of subungual pigmentation.^7^ The recommended treatment for subungual venous hemangioma is surgical excision, as other reported treatments for venous hemangiomas, such as cryosurgery, electrosurgery, sclerotherapy, infrared coagulation, and laser therapy are impractical for subungual venous hemangiomas given the overlying protective nail plate.^4^^,^^5^

## Conflicts of interest

None disclosed.
